# ﻿The systematic position of *Cryptopotamonanacoluthon* (Kemp, 1918), with the description of a new species of *Sinolapotamon* Tai & Sung, 1975 (Crustacea, Decapoda, Brachyura, Potamidae) from southern China

**DOI:** 10.3897/zookeys.1166.101737

**Published:** 2023-06-08

**Authors:** Yuan-Biao Lu, Yi-Xuan Zhang, Jie-Xin Zou

**Affiliations:** 1 Research Laboratory of Freshwater Crustacean Decapoda & Paragonimus, School of Basic Medical Sciences, Nanchang University, Nanchang City, Jiangxi Province, China Nanchang University Nanchang City China; 2 Jiangxi Provincial Key Laboratory of Experimental Animals, Nanchang City, Jiangxi Province, China Jiangxi Provincial Key Laboratory of Experimental Animals Nanchang City China

**Keywords:** Freshwater crabs, molecular phylogeny, systematics, taxonomy

## Abstract

The systematics of the potamid freshwater crab *Cryptopotamonanacoluthon* (Kemp, 1918) is clarified, and its generic position in *Sinolapotamon* Tai & Sung, 1975, is confirmed based on morphological comparisons, geographical information and phylogenetic analyses. A new species of *Sinolapotamon*, *Sinolapotamoncirratum***sp. nov.** is described from the Guangxi Zhuang Autonomous Region of China. *Sinolapotamoncirratum***sp. nov.** is distinguished from its congeners by the combination of characters of its carapace, third maxilliped, anterolateral margin, and unique male first gonopod. Phylogenetic analyses based on partial COX1, 16S rRNA and 28S rRNA genes also support the species as new.

## ﻿Introduction

**Figure 1.**
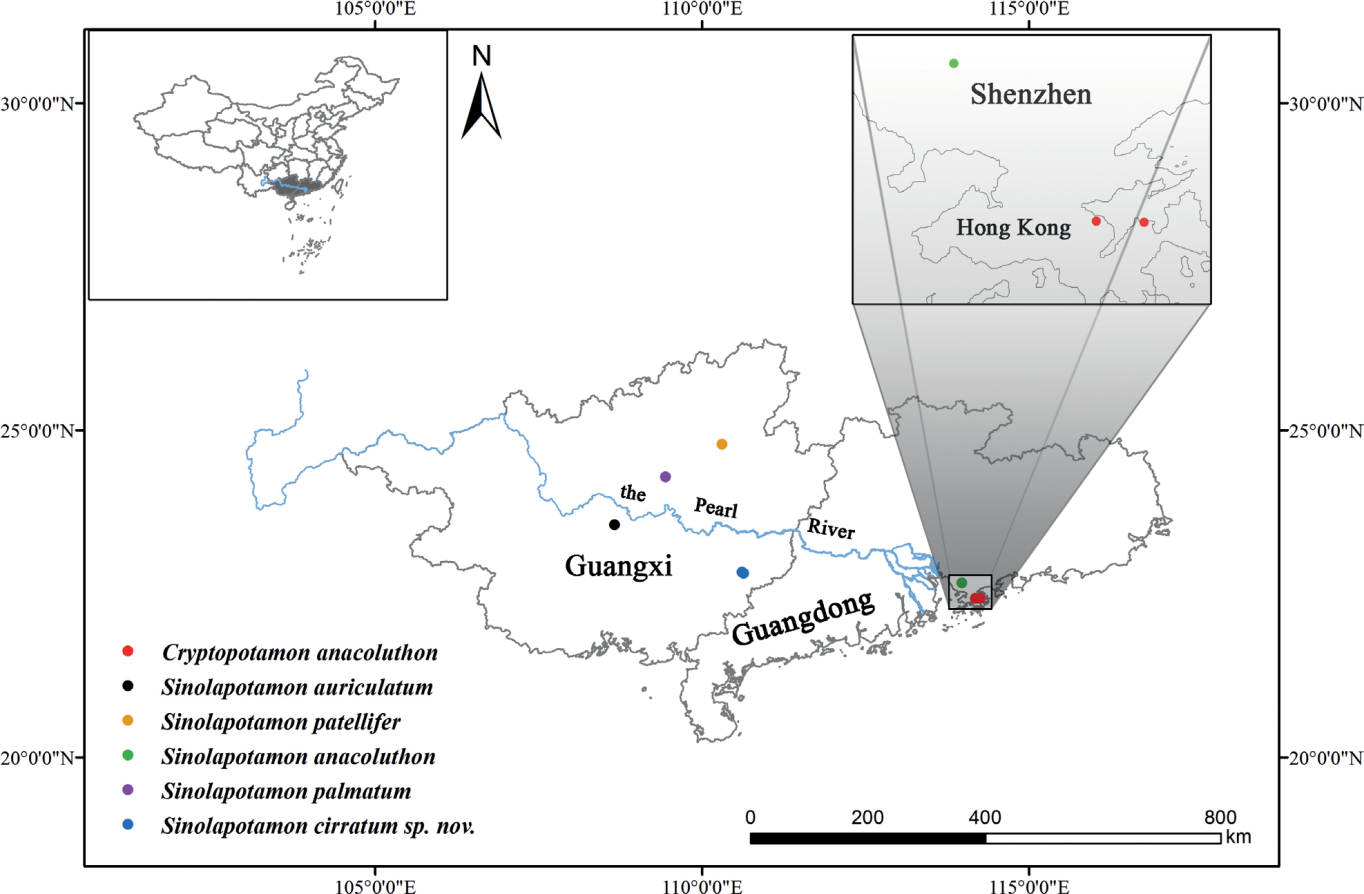
Map showing collection sites of *Sinolapotamon* used in this study and the known collection sites of *Cryptopotamonanacoluthon.* Part of the studied region (Shenzhen and Hong Kong) is enlarged.

Located in the southwest border region of China, with a warm climate, abundant precipitation, and a high percentage of forest coverage and karst landforms, Guangxi (Fig. 1) provides a suitable living environment for freshwater crabs. In China, which has the highest species richness of freshwater crabs globally (Cumberlidge et al. 2011), the species richness in Guangxi (43 species, including *S.cirratum* sp. nov.) is surpassing that of Taiwan (41 species) and only lower than that of Yunnan (74 species) (Chu et al. 2018; Wang et al. 2019; Cai et al. 2021). Rong County, situated in southeastern Guangxi and adjoining Guangdong Province, is the type locality of *Sinolapotamoncirratum* sp. nov. (Fig. 1). Hong Kong (Fig. 1), located in the south of China, consists of Hong Kong Island, Kowloon, the New Territories and 262 surrounding islands. The New Territories and Kowloon are connected to mainland China. It is worth noting that the New Territories is connected to Shenzhen, Guangdong Province.

The genus *Sinolapotamon* Tai & Sung, 1975, was described, with the type species as Potamon (Geothelphusa) patellifer Wu, 1934 (type locality, Luocheng). Zhu et al. (2010) described two species of *Sinolapotamon* from Guangxi: *S.auriculatum* Zhu, Naruse & Zhou, 2010, from Shanglin, and *S.palmatum* Zhu, Naruse & Zhou, 2010, from Liuzhou (Fig. 1). There has been disagreement regarding the generic position of *Cryptopotamonanacoluthon* (Kemp, 1918). Ng and Dudgeon (1992), while describing *Cryptopotamon* Ng & Dudgeon, 1992, included only Potamon (Potamon) anacoluthon Kemp, 1918, from Hong Kong (Fig. 1). Dai (1999) pointed out that *Cryptopotamon* is a synonym of *Sinolapotamon*, considering that the differences between these two genera could only be regarded as interspecific differences. Ng et al. (2008), however, continued to place Potamon (Potamon) anacoluthon in *Cryptopotamon*. Based on phylogenetic methods, we reconstructed the phylogenetic relationships of *Sinolapotamon*, which confirm the generic position of Potamon (Potamon) anacoluthon in *Sinolapotamon* and recognize a new species. *Sinolapotamoncirratum* sp. nov. is described here based on morphology and genetic data.

## ﻿Materials and methods

Specimens were collected from the Duqiaoshan Forest Park and Silaochong, both in Rong County, Yulin City, Guangxi Zhuang Autonomous Region, China. The two sites are so close that they appear as one dot in Fig. 1 (about 5 km). In addition, the sites of the specimens of *C.anacoluthon* referred to in Ng and Dudgeon (1992) were added to the map (Fig. 1). The two sites are: Tai Po Kau Forest Reserve stream, New Territories, Hong Kong; and the stream at Wu Kwai Sha, New Territories, Hong Kong. The linear distances from the ‘Shenzhen’ site to the two ‘Hong Kong’ sites are between 30–40 km (Fig. 1). Ethanol (95%) was used to preserve the collected specimens, which were deposited in the Department of Parasitology of the Medical College of the Nanchang University, Jiangxi, China (NCU MCP). Materials used herein, except for the new species and *S.anacoluthon*, are as follows: *S.patellifer*, 1 ♂, Yangshuo County, Guangxi Province, collection date not clear, NCU MCP 407301; *S.auriculatum*, 2 ♂♂, Shanglin County, Guangxi Province, July 2006, NCU MCP 72301, 72302; *S.palmatum*, 2 ♂♂, Liuzhou City, Guangxi Province, May 2018, NCU MCP 415301, 415302. Carapace width and length were measured in millimeters. The terminology used herein primarily follows that of Dai (1999) and Davie et al. (2015). The abbreviations used for the male first gonopod and male second gonopod are G1 and G2, respectively.

Approximately 50 mg of muscle tissue was excised from ambulatory legs. Total genomic DNA was extracted using the D3373-01 Mollusc DNA Kit (Omega Biotek, Inc., Norcross, USA). In our study, three fragments of target genes were amplified, including the mitochondrial COX1 and 16S rRNA genes and nuclear 28S rRNA gene. The primers and annealing temperatures used are presented in Table 1. Notably, the COX1 primers used were slightly modified based on the primers LCO1490 and HCO2198. A base T in the primer HCO2198 was replaced with a degenerate base Y (Folmer et al. 1994; Yang 2011). We performed phylogenetic analyses with the single-gene dataset (COX1) and 3-gene combined dataset (COX1, 16S rRNA and 28S rRNA). All molecular data are presented in Table 2. Sequences were aligned using ClustalW (Thompson et al. 2003), and the conserved regions were selected with Gblocks 0.91b (Castresana 2000) using the default settings. The optimal model for Bayesian inference (BI) analysis was determined using MrModeltest v. 2.3 (Nylander 2004) on the basis of the Akaike information criterion (AIC). The best-fitting model was GTR+G+I for both datasets. MrBayes v. 3.2.6 (Ronquist et al. 2012) was employed to perform BI analysis, and four Monte Carlo Markov chains of 2 000 000 generations were run with sampling every 1000 generations. The first 25% of generations were discarded as burn-in. Tracer v. 1.6 (Rambaut et al. 2013) was used to examine the sampling parameter. The optimal model, identified with MEGA X, for maximum likelihood (ML) analysis was also GTR+G+I for both datasets (Kumar et al. 2018). MEGA X was also employed to construct the ML tree based on 1000 bootstrap replicates and to calculate the pairwise distance based on the Kimura 2-parameter (K2P) model (Kumar et al. 2018). The map of the study area was prepared using ArcMap v. 10.2.

**Table 1.** Information on the primers used and annealing temperatures.

Gene	Primer name	Sequence 5’-3’	Product Length (bp)	Annealing Temperature (°C)	Reference
COI	LCO1490	GGTCAACAAATCATAAAGATATTGG	~700	52	Folmer et al. (1994) and Yang (2011)
	HCO2198’	TAAACTTCAGGGTGACCAAAAAAYCA			
16S rRNA	1471	CCTGTTTANCAAAAACAT	~600	50	Crandall and Fitzpatrick (1996)
	1472	AGATAGAAACCAACCTGG			
28S rRNA	F28S	CAGCCCTAAGCAGGTGGTAAACT	~1000	53	Ji et al. (2016)
	R28S	CCATCTTTCGGGTCCCAACAT			

**Table 2.** Collection information and GenBank accession number of the species used for phylogenetic analyses.

Species	GenBank number	Voucher number	Locality	Reference
*Candidiopotamonokinawense* Minei, 1973	COI, MN737145	–	Okinawa, Japan	Zhang et al. 2020
*Candidiopotamonrathbunae* (De Man, 1914)	COI, AB433579	NCHU:ZOOL:13146	Taiwan	Shih et al. 2011a
*Chinapotamondepressum* (Dai, Song, Li & Liang, 1980)	COI, MZ350918	–	Guangdong, China	Pan et al. 2021
*Geothelphusadehaani* (White, 1847)	COI, AB187570	–	Tokyo, Japan	Segawa and Aotsuka 2005
*Geothelphusaminei* Shy & Ng, 1998	COI, AB625725	Gmn6	Ryukyu	Shih et al. 2011b
*Huananpotamonangulatum* (Dai, Chen, Song, Fan, Lin & Zeng, 1979)	COI, AB433576	NCHU:ZOOL:13139	Fujian, China	Shih et al. 2011b
*Nanhaipotamonguangdongense* Dai, 1997	COI, MK226144	Ns7	Guangdong, China	Huang et al. 2018
*Nanhaipotamonhongkongense* (Shen, 1940)	COI, AB470509	Nh3	Hong Kong	Shih et al. 2011b
*Nanhaipotamonpingyuanense* Dai, 1997	COI, AB470513	Npy3	Guangdong, China	Shih et al. 2011b
*Neotiwaripotamonjianfengense* Dai & Naiyanetr, 1994	COI, MZ350933	–	Hainan, China	Pan et al. 2021
*Sinopotamonyaanense* (Chung & Ts’ao, 1962)	COI, LC155173	SC8	Sichuan, China	Shih et al. 2016
*Longpotamonnanlingense* (Dai & Jiang, 1991)	COI, LC155196	SPx173	Hunan, China	Shih et al. 2016
*Cantopotamonzhuhaiense* Huang, Ahyong & Shih, 2017	COI, LC342051	SYSBM:1439	Guangdong, China	Huang et al. 2017
*Parapotamonspinescens* (Calman, 1905)	COI, LC155209	PP4	Yunnan, China	Shih et al. 2016
*Tenuilapotamonlatilum* (Chen, 1980)	COI, LC155206	TNL1	Hubei, China	Shih et al. 2016
*Tiwaripotamonedostilus* Ng & Yeo, 2001	COI, AB896762	TWs6	Haiphong, Vietnam	Shih and Do 2014
*Tiwaripotamonpingguoense* Dai & Naiyanetr, 1994	COI, LC145315	TWs13	Guangxi, China	Van et al. 2016
*Yarepotamongracillipa* (Dai, Song, Li & Liang, 1980)	COI, AB433577	–	Guangxi, China	Direct Submission
*Apotamonauteshainanensis* (Parisi, 1916)	COI, MN737137	–	Hainan, China	Zhang et al. 2020
*Chinapotamonmaolanense* Zou, Bai & Zhou, 2018	COI, MT134100	–	Guizhou, China	Cui et al. 2020
*Indochinamonchinghungense* (Dai, Song, He, Cao, Xu & Zhong, 1975)	COI, MZ350925	–	Yunnan, China	Pan et al. 2021
*Indochinamondaweishanense* (Dai, 1995)	COI, MZ350926	–	Yunnan, China	Pan et al. 2021
*Potamiscusyiwuensis* Dai & Cai, 1998	COI, MN737136	–	Yunnan, China	Zhang et al. 2020
*Qianguimonelongatum* Huang, 2018	COI, MZ350943	–	Guizhou, China	Pan et al. 2021
*Vadosapotamonsheni* (Dai & Chen in Dai, Chen, Liu, Luo, Yi, Liu, Gu & Liu, 1990)	COI, MZ350958	–	Sichuan, China	Pan et al. 2021
*Longpotamonyangtsekiense* (Bott, 1967)	COI, EU676302	TB5	China	Direct Submission
*Tenuilapotamonjoshuiense* (Dai, Song, He, Cao, Xu & Zhong, 1975)	COI, MZ350951	–	Hunan, China	Pan et al. 2021
*Neotiwaripotamonwhiteheadi* (Parisi, 1916)	COI, MZ350934	–	Hainan, China	Pan et al. 2021
*Huananpotamonlichuanense* Dai, Zhou & Peng, 1995	COI, MN737141	–	Jiangxi, China	Zhang et al. 2020
*Johorasingaporensis* Ng, 1986	COI, MG010237	JSIN_BTM01	Singapore	Tay et al. 2018
*S.cirratum* sp. nov.	COI, OP425670	Slsp01	Guangxi, China	This study
	COI, OP425672	Slsp02	Guangxi, China	This study
	COI, OP425671	Slsp03	Guangxi, China	This study
*S.auriculatum* Zhu, Naruse & Zhou, 2010	COI, OP425667	Slac01	Guangxi, China	This study
	COI, OP376822	Slpm02	Guangxi, China	This study
*S.anacoluthon* (Kemp, 1918)	COI, OP425668	Slal01	Guangdong China	This study
	COI, OP425669	Slal02	Guangdong China	This study
*S.patellifer* (Wu, 1934)	COI, MK883709	–	Guangxi, China	Ji et al. 2019
*Candidiopotamonokinawense* Minei, 1973	16S, AB208627	Co	Okinawa, Japan	Shih et al. 2006
*Candidiopotamonrathbunae* (De Man, 1914)	16S, AB208589	TPWL1	Taiwan	Shih et al. 2006
*Chinapotamondepressum* (Dai, Song, Li & Liang, 1980)	16S, KT586287	–	Guangxi, China	Ji et al. 2016
*Geothelphusadehaani* (White, 1847)	16S, AB535460	Gd21	Kagoshima, Japan	Ng et al. 2010
*Geothelphusaminei* Shy & Ng, 1998	16S, AB625677	Gmn8	Ryukyu	Shih et al. 2011b
*Huananpotamonangulatum* (Dai, Chen, Song, Fan, Lin & Zeng, 1979)	16S, AB433555	NCHU:ZOOL:13139	Fujian, China	Shih et al. 2011b
*Nanhaipotamonhongkongense* (Shen, 1940)	16S, AB212869	NHHK	Hong Kong	Shih et al. 2005
*Nanhaipotamonpingyuanense* Dai, 1997	16S, AB265237	NPy	Guangdong, China	Shih et al. 2007
*Neotiwaripotamonjianfengense* Dai & Naiyanetr, 1994	16S, KT586289	–	Hainan, China	Ji et al. 2016
*Sinopotamonyaanense* (Chung & Ts’ao, 1962)	16S, KT586263	02	Sichuan, China	Ji et al. 2016
*Longpotamonnanlingense* (Dai & Jiang, 1991)	16S, KT586180	01	Hunan, China	Ji et al. 2016
*Tenuilapotamonlatilum* (Chen, 1980)	16S, AB428468	–	Hubei, China	Shih et al. 2009
*Longpotamonyangtsekiense* Bott, 1967	16S, KT586268	02	Jiangsu, China	Ji et al. 2016
*Tenuilapotamonjoshuiense* (Dai, Song, He, Cao, Xu & Zhong, 1975)	16S, ON024657	NCU MCP 430301	Hunan, China	Direct Submission
*S.cirratum* sp. nov.	16S, OP467587	Slsp01	Guangxi, China	This study
	16S, OP467588	Slsp02	Guangxi, China	This study
	16S, OP467584	Slsp03	Guangxi, China	This study
*S.auriculatum* Zhu, Naruse & Zhou, 2010	16S, OP467583	Slac01	Guangxi, China	This study
	16S, OP467590	Slpm02	Guangxi, China	This study
*S.anacoluthon* (Kemp, 1918)	16S, OP467585	Slal01	Guangdong, China	This study
	16S, OP467586	Slal02	Guangdong, China	This study
*S.patellifer* (Wu, 1935)	16S, MK883709	–	Guangxi, China	Ji et al. 2019
*Candidiopotamonokinawense* Minei, 1973	28S, AB503625	Co	Okinawa, Japan	Direct Submission
*Candidiopotamonrathbunae* (De Man, 1914)	28S, AB503628	Cr	Taiwan	Direct Submission
*Chinapotamondepressum* (Dai, Song, Li & Liang, 1980)	28S, KT586427	–	Guangxi, China	Ji et al. 2016
*Geothelphusadehaani* (White, 1847)	28S, AB503607	Gdmms	Kagoshima, Japan	Direct Submission
*Geothelphusaminei* Shy & Ng, 1998	28S, AB503619	GmnIG	Okinawa, Japan	Direct Submission
*Huananpotamonangulatum* (Dai, Chen, Song, Fan, Lin & Zeng, 1979)	28S, AB576807	Hua2	Fujian, China	Shih et al. 2011b
*Nanhaipotamonhongkongense* (Shen, 1940)	28S, AB551401	Nh3	Hong Kong	Shih et al. 2011b
*Nanhaipotamonpingyuanense* Dai, 1997	28S, AB551405	Npy2	Guangdong, China	Shih et al. 2011b
*Neotiwaripotamonjianfengense* Dai & Naiyanetr, 1994	28S, KT586429	–	Hainan, China	Ji et al. 2016
*Sinopotamonyaanense* (Chung & Ts’ao, 1962)	28S, KT586416	04	Sichuan, China	Ji et al. 2016
*Longpotamonnanlingense* (Dai & Jiang, 1991)	28S, KT586368	01	Hunan, China	Ji et al. 2016
*Tenuilapotamonlatilum* (Chen, 1980)	28S, MW540828	NCU MCP 66301	Hubei, China	Direct Submission
*Longpotamonyangtsekiense* (Bott, 1967)	28S, KT586417	02	Jiangsu, China	Ji et al. 2016
*Tenuilapotamonjoshuiense* (Dai, Song, He, Cao, Xu & Zhong, 1975)	28S, ON033004	NCU MCP 430301	Hunan, China	Direct Submission
*S.cirratum* sp. nov.	28S, OP578215	Slsp01	Guangxi, China	This study
	28S, OP578219	Slsp02	Guangxi, China	This study
	28S, OP578212	Slsp03	Guangxi, China	This study
*S.auriculatum* Zhu, Naruse & Zhou, 2010	28S, OP578218	Slac01	Guangxi, China	This study
	28S, OP578217	Slpm02	Guangxi, China	This study
*S.anacoluthon* (Kemp, 1918)	28S, OP578213	Slal01	Guangdong, China	This study
	28S, OP578214	Slal02	Guangdong, China	This study
*S.patellifer* (Wu, 1936)	28S, OP578216	Slpl01	Guangxi, China	This study

## ﻿Results

### ﻿Systematics

**Family Potamidae Ortmann, 1896**

#### ﻿ Sinolapotamon

Taxon classificationAnimaliaDecapodaPotamidae

Tai & Sung, 1975

DF39E753-39F1-5F5C-A447-F989A753ACE0

Cryptopotamon Ng & Dudgeon, 1992: 741, figs 3B, 4, 5.

##### Type species.

Potamon (Geothelphusa) patellifer Wu, 1934, by original designation.

#### ﻿ Sinolapotamon anacoluthon

Taxon classificationAnimaliaDecapodaPotamidae

(Kemp, 1918)

67C45D86-5B9A-5924-A67F-0302904ED4F6

Figs 2 , 7C

Potamon (Potamon) anacoluthon Kemp, 1918: 243, fig. 5.
Cryptopotamon anacoluthon Ng & Dudgeon, 1992: 741, figs 3B, 4, 5. — Ng et al. 2008: 161 (list).
Sinolapotamon anacoluthon Dai, 1999: 150, fig. 79.

##### Material examined.

China • 4 ♂♂ (18.40 × 16.34 mm, 20.26 × 18.40 mm, 21.64 × 18.60 mm, 19.26 × 17.04 mm); Yangtaishan Forest Park, Shenzhen, Guangdong Province; 22.6587°N, 113.9837°E; July 2022; Sheng Yu leg.; NCU MCP 434001–434004 • 1 ♂ (25.84 × 22.76 mm); same collection data as above; NCU MCP 434101 • 3 ♀♀ (26.34 × 23.58 mm, 28.84 × 24.38 mm, 24.31 × 20.95 mm); same collection data as above; NCU MCP 434102–434104.

##### Diagnosis.

**Figure 2.**
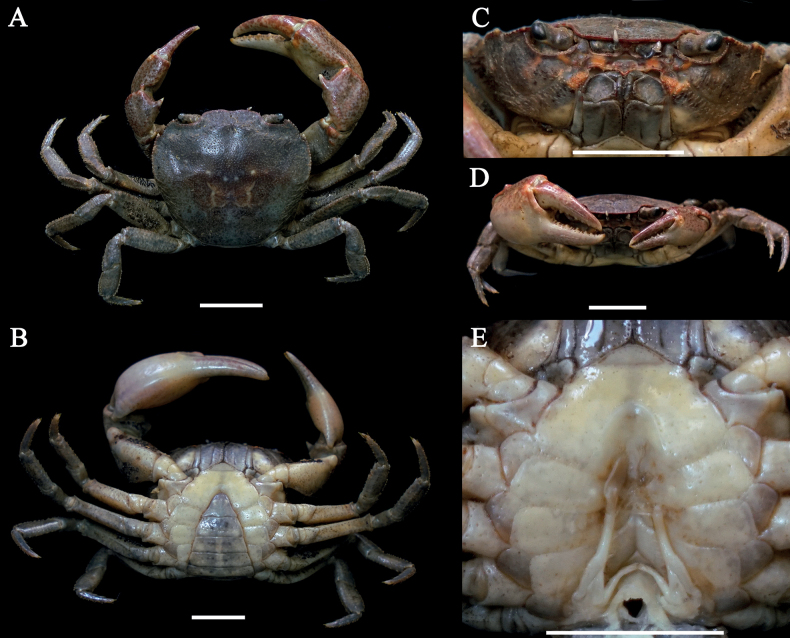
*Sinolapotamonanacoluthon*, male (25.84 × 22.76 mm) (NCU MCP 434101) **A** overall dorsal habitus **B** overall ventral habitus **C** frontal view of cephalothorax **D** outer view of chelipeds **E** sternopleonal cavity with G1. Scale bars: 1 cm.

Carapace gently convex, regions indistinct. Cervical groove shallow, indistinct; H-shaped groove depressed and distinct (Fig. 2A). Epigastric cristae weak, postorbital cristae flat, indistinct. External orbital angle triangular, with about 5 small granules. Epibranchial tooth sharp, distinctly separated with external orbital angle by V-shaped gap. Anterolateral margin of carapace cristate, with about 12 granules (Fig. 2A). Maxilliped 3 exopod reaching nearly 1/3 of merus length, with long flagellum (Fig. 2C). Chelipeds (pereiopod 1) strongly unequal (Fig. 2A, B, D). G1 slender, subterminal segment about 1.1 times as long as terminal segment; 2 lobes of terminal segment strongly unequal, dorsal lobe longitudinally extended, oval shaped, ventral lobe sharp and short, reaching 3/7 of terminal segment (Figs 2E, 7C).

##### Ecology.

The species is usually inhabiting the clear hill streams at an altitude below 50 m. Stones could serve as shelter and leaf mould could serve as food (Dai, 1999).

##### Distribution.

China: Shenzhen of Guangdong Province (present record) and Hong Kong.

##### Remarks.

The specimens from Shenzhen, with gently convex dorsal surface of carapace, indistinct postorbital cristae, sharp epibranchial tooth, unequal lobes of the terminal segment of the G1 (Fig. 2), and other characteristics, agree well with the descriptions and illustrations in Ng and Dudgeon (1992) and Dai (1999). The ratio of the subterminal segment to the terminal segment of G1 calculated in this study is 1.1 (Fig. 7C), which is equal to that in Dai (1999) and slightly smaller than that in Ng and Dudgeon (1992) (1.17). Although the specimens are not from Hong Kong, they could still be determined as *S.anacoluthon* based on morphological examination and the proximity of their collection site to Hong Kong (Fig. 1).

**Figure 3.**
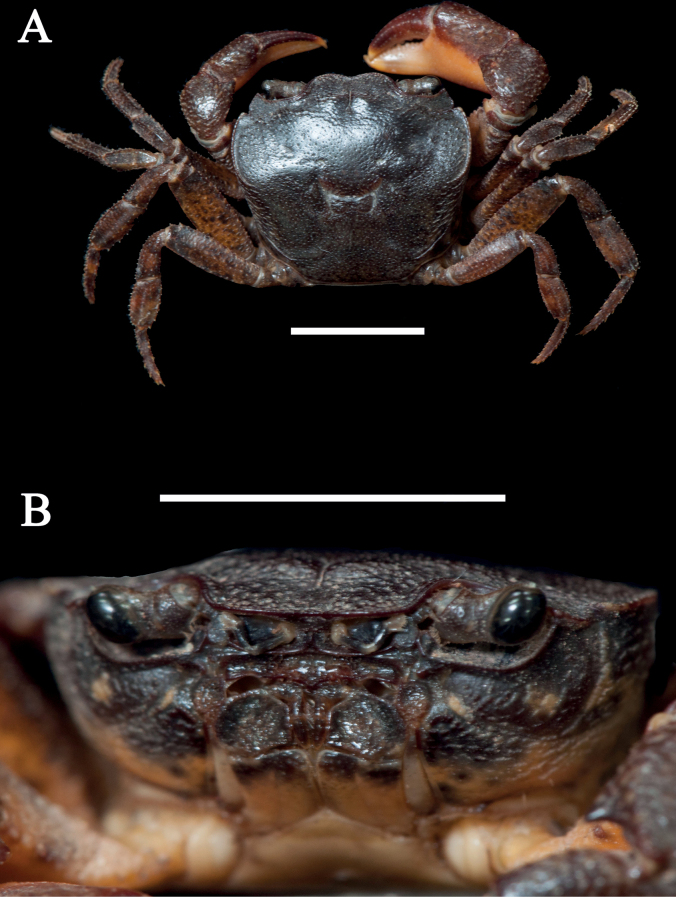
*Sinolapotamoncirratum* sp. nov., holotype male (17.90 × 15.50 mm) (NCU MCP 434201) **A** overall dorsal habitus **B** frontal view of cephalothorax. Scale bars: 1 cm.

Ng and Dudgeon (1992) listed the differences between *Cryptopotamon* and *Sinolapotamon*, including carapace, epigastric cristae, postorbital cristae, epibranchial tooth, and the ratio of the subterminal segment to the terminal segment of the G1. We, however, noticed that those differences are interspecific, while two or more species sharing the same character state with the remaining species is not. For instance, *S.anacoluthon* has a gently convex carapace similar to that of *S.cirratum* sp. nov. but different from the remaining congeners (Figs 2A, 3A). The weak epigastric cristae of *S.anacoluthon* are consistent with those of *S.auriculatum* and *S.cirratum* sp. nov. (Fig. 2A; see Zhu et al. 2010: figs 1a, 6a). The indistinct postorbital cristae of *S.anacoluthon* are comparable with those of *S.auriculatum* and *S.palmatum* (Fig. 2A; see Zhu et al. 2010: figs 1a, 6a). The sharp epibranchial tooth is consistent with that of *S.auriculatum* (Fig. 2A; see Zhu et al. 2010: fig. 1a). The different ratios of the subterminal segment to the terminal segment of the G1 could only be regarded as interspecific differences. Most importantly, all five species have accordant fundamental types of G1 (Fig. 7).

#### ﻿ Sinolapotamon cirratum sp. nov.

Taxon classificationAnimaliaDecapodaPotamidae

6582241E-C662-50D6-B9E6-86CAF90C8FA4

https://zoobank.org/25AEC22F-BAAA-4DF5-8580-8334F8DBB9EC

**Figure 4.**
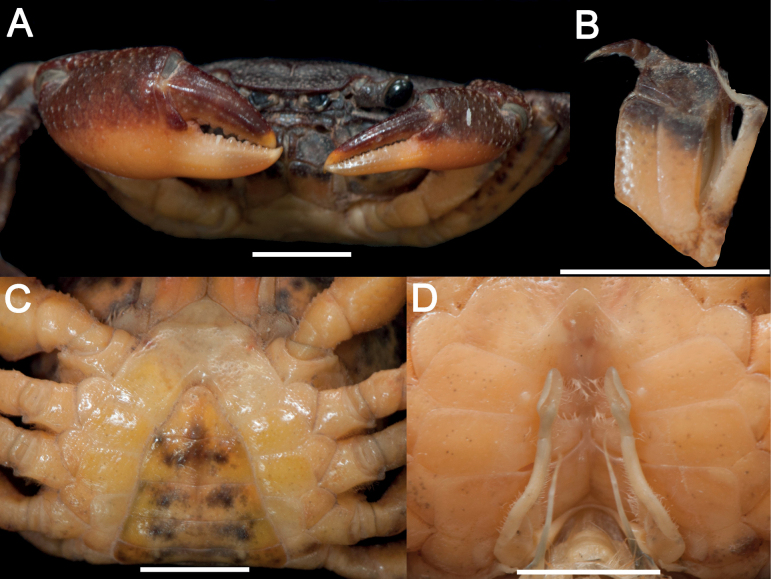
*Sinolapotamoncirratum* sp. nov., holotype male (17.90 × 15.50 mm) (NCU MCP 434201) **A** outer view of chelipeds **B** left third maxilliped **C** anterior thoracic sternum, pleonal somites 4–6 and telson **D** sternopleonal cavity with G1. Scale bars: 5 mm.

**Figure 5.**
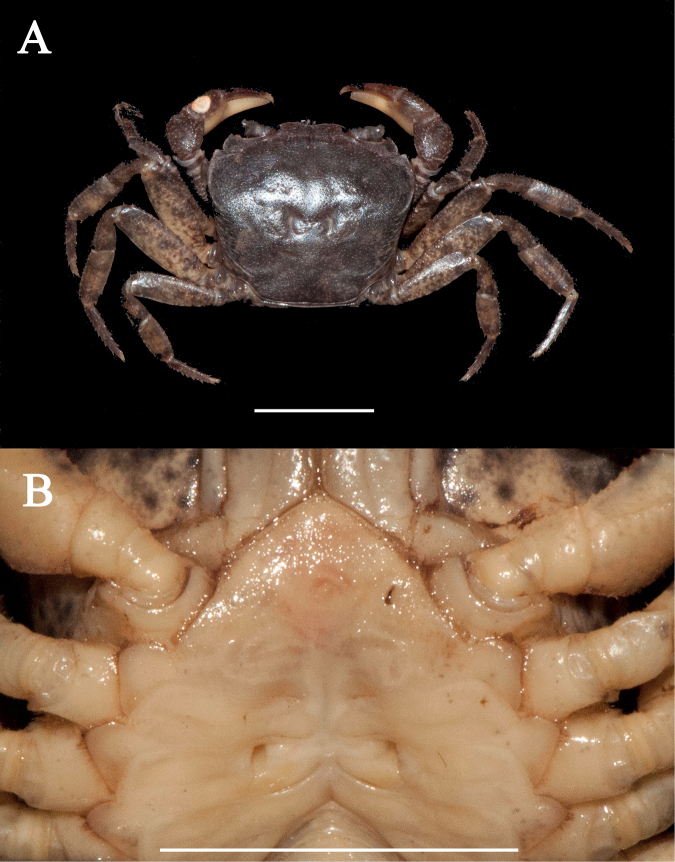
*Sinolapotamoncirratum* sp. nov., paratype female (17.42 × 15.45 mm) (NCU MCP 433901) **A** overall dorsal habitus **B** sternopleonal cavity with vulvae. Scale bars: 1 cm.

**Figure 6.**
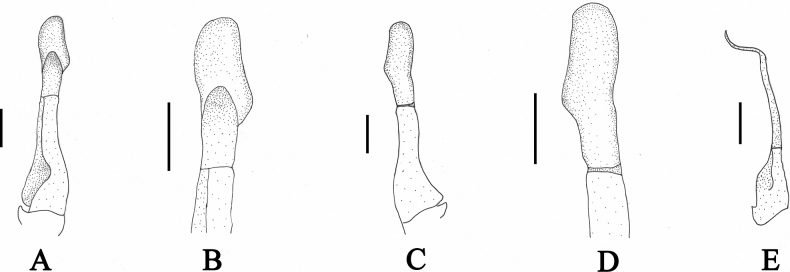
Gonopods of *Sinolapotamoncirratum* sp. nov., holotype male (17.90 × 15.50 mm) (NCU MCP 434201) **A** ventral view of left G1 **B** ventral view of terminal segment of left G1 **C** dorsal view of left G1 **D** dorsal view of terminal segment of left G1 **E** ventral view of left G2. Scale bars: 1 mm.

**Figure 7.**
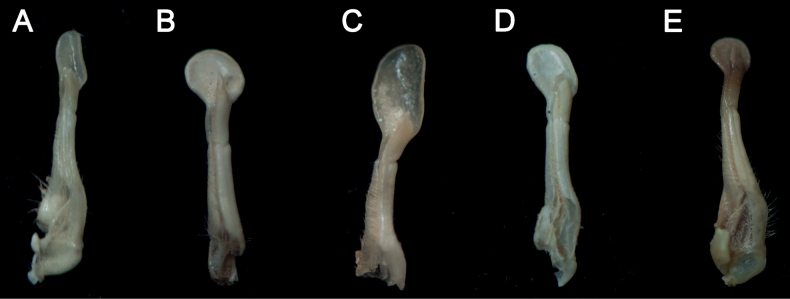
Left G1s (ventral view) of known species of *Sinolapotamon***A***Sinolapotamoncirratum* sp. nov., holotype (NCU MCP 434201) **B***S.patellifer* (Yangshuo, NCU MCP 407301) **C***S.anacoluthon* (Shenzhen, NCU MCP 434001) **D***S.auriculatum* (Shanglin, NCU MCP 72301) **E***S.palmatum* (Liuzhou, NCU MCP 415301).

**Figure 8.**
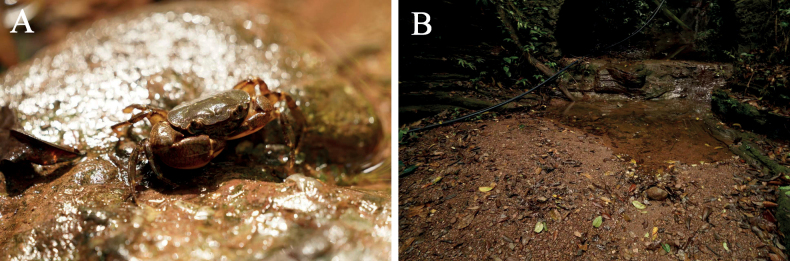
**A***Sinolapotamoncirratum* sp. nov. in the wild **B** general habitat of *Sinolapotamoncirratum* sp. nov.

Figs 3 , 4 , 5 , 6 , 7A , 8

##### Type material.

***Holotype***: China • ♂ (17.90 × 15.50 mm); Guangxi Zhuang Autonomous Region, Yulin City, Rong County, Duqiaoshan Forest Park; 22.8019°N, 110.6098°E; October 2022; Yi-Xuan Zhang leg.; NCU MCP 434201. ***Paratypes***: CHINA • 1 ♀ (17.42 × 15.45 mm); same collection data as for holotype; NCU MCP 433901 • 1 ♀ (23.74 × 20.30 mm); same collection data as for holotype; NCU MCP 433902 • 4 ♂♂ (22.54 × 19.55 mm, 22.92 × 19.80 mm, 23.05 × 20.24 mm, 18.00 × 16.02 mm); same collection data as for holotype; NCU MCP 433903–433906.

##### Other material.

China • 7 ♂♂ (21.96 × 19.12 mm, 13.36 × 11.93 mm, 15.76 × 13.18 mm, 17.34 × 14.99 mm, 17.26 × 14.94 mm, 17.93 × 14.91 mm, 20.73 × 17.62 mm); same collection data as for holotype; NCU MCP 434202–434208 • 8 ♂♂ (14.99 × 12.90 mm, 9.48 × 8.65 mm, 9.61 × 8.54 mm, 9.81 × 8.91 mm, 8.71 × 7.53 mm, 8.82 × 7.77 mm, 9.74 × 7.38 mm, 10.42 × 8.79 mm); Guangxi Zhuang Autonomous Region, Yulin City, Rong County, Silaochong, small stream; 22.8263°N, 110.6065°E; November 2018; Jie-Xin Zou et al. leg.; NCU MCP 416001–416008.

##### Diagnosis.

Carapace subquadrate, regions indistinct; dorsal surface gently convex, anterolateral region weakly rugose. Cervical groove shallow and wide; H-shaped groove shallow (Figs 3A, 5A). Epigastric cristae distinct, separated from postorbital cristae by narrow gap; epibranchial region slightly depressed; mesogastric region gently convex. External orbital angle triangular, distinctly separated from anterolateral margin by wide notch. Anterolateral margin of carapace distinctly cristate, lined with approximately 20 granules (Figs 3A, 5A). Maxilliped 3 exopod reaching nearly 1/2 of merus length, with long flagellum, slightly longer than width of merus (Fig. 4B). Chelipeds (pereiopod 1) strongly unequal in males, subequal in females (Figs 3A, 4A, 5A). G1 slender, subterminal segment about 1.7 times as long as terminal segment; 2 lobes of terminal segment strongly unequal, dorsal lobe longitudinally extended, oval shaped, ventral lobe blunt, reaching 1/2 of terminal segment (Figs 6A–D, 7A). Female vulvae ovate, medium-sized, occupying anterior 2/3 length of sternite 6 (Fig. 5B).

##### Description.

Carapace subquadrate, nearly 1.2 times as wide as long; surface generally smooth, regions indistinct; dorsal surface slightly convex, with tiny pits, anterolateral region weakly rugose. Cervical groove shallow and wide; H-shaped groove shallow. Front gently deflexed; frontal margin slightly rimmed, weakly bilobed in dorsal view (Figs 3A, 5A). Epigastric cristae low and weak, separated from postorbital cristae by narrow gap; postorbital cristae laterally expanded, not fused with epibranchial tooth. Epibranchial region gently depressed; mesogastric region gently convex. External orbital angle bluntly triangular, distinctly separated from anterolateral margin by V-shaped wide gap (Figs 3A, 5A). Epibranchial tooth distinct, bluntly angular. Anterolateral margin of carapace distinctly cristate, lined with approximately 20 granules; bent inward posteriorly. Posterolateral surface smooth, with oblique striae, converging towards posterior carapace margin (Figs 3A, 5A). Orbits large; supraorbital and infraorbital margins cristate. Sub-orbital, pterygostomial, subhepatic regions covered with striae. Epistome posterior margin narrow longitudinally; median lobe triangular, lateral margins sinuous (Fig. 3B).

Maxilliped 3 exopod reaching nearly 1/2 of merus length, with long flagellum, slightly longer than width of merus. Merus subrectangular, 2 times as wide as long. Ischium subtrapezoidal, about 1.4 times as long as wide, with distinct sulcus (Fig. 4B).

Chelipeds (pereiopod 1) strongly unequal in males, subequal in females. Merus trigonal in cross section. Carpus surface gently depressed, with spine at inner distal angle and spinule at base in both males and females. Palm of lager chela about 1.3–1.5 times as long as high in males, 1.3–1.6 times in females. Dactylus of larger chela 0.6–1.0 times as long as palm in males, practically same proportion in females. Inner margin of fingers lined with granular teeth; fingers of lager chela leaving small gap while smaller one without gap when closed in both males and females (Figs 3A, 4A, 5A).

Ambulatory legs (pereiopod 2–5) slender, with setae; pereiopod 3 longest, merus 0.5–0.6 times as long as carapace length. Pereiopod 5 propodus about 2.0 times as long as broad in both males and females, 0.8–0.9 times as long as dactylus; dactylus gently curved (Figs 3A, 5A).

Male thoracic sternum generally smooth, pitted; sternite 1 triangular; sternite 2–3 fused without visible sutures. Male sternopleonal cavity relatively deep, exceeding imaginary line connecting posterior edges of cheliped coxae base. Median longitudinal suture of sternites 7, 8 deep. Tubercle of abdominal lock positioned at approaching mid-length of sternite 5. Sutures between sternites in female indistinct (Fig. 4C, D). Female vulvae ovate, medium-sized, occupying anterior 2/3 length of sternite 6 (Fig. 5B).

Male pleon and telson triangular; pleonal somites 3–6 gradually narrowed longitudinally, lateral margins forming gently concave line with thoracic sternum; pleonal somite 6 about 2.2 times as wide as long; telson about 1.3 times as wide as long (Fig. 4C). Female pleon and telson broadly ovate (Fig. 5B).

G1 slender, tip of terminal segment exceeding beyond pleonal locking tubercle and suture between thoracic sternites 4 and 5 (Fig. 4D). Subterminal segment about 1.7 times as long as terminal segment; edges of dorsal lobe curled; 2 lobes of terminal segment strongly unequal, dorsal lobe longitudinally extended, oval, ventral lobe blunt, reaching 1/2 length of terminal segment (Fig. 6A–D). G2 slender, longer than G1 (Fig. 6A, E).

##### Remarks.

Consistent with the diagnostic characters of *Sinolapotamon*, *Sinolapotamoncirratum* sp. nov. has a gently convex dorsal surface, long flagellum of the third maxilliped exopod and unequal lobes of the G1 terminal segment (Figs 3A, 4B, 7A). The dorsal lobe of the G1 terminal segment in *S.cirratum* sp. nov. is long and oval, which is similar to that of *S.anacoluthon*. The two species can nevertheless be distinguished by the ratio of the subterminal segment to the terminal segment of G1, which is 1.7 in *S.cirratum* sp. nov. and 1.1 in *S.anacoluthon* (Fig. 7A, C). When compared with *S.patellifer*, *S.auriculatum* and *S.palmatum*, the new species could be easily distinguished by the shape of the dorsal lobes and ventral lobes. The ventral lobe of *S.cirratum* sp. nov. is bluntly angular, while those of the other species in *Sinolapotamon* are pointed or shortly pointed (Fig. 7A, B, D, E). They also differ in comparative length of the ventral lobe relative to the terminal segment of the G1 (see Table 3). Additional differences among the known species of *Sinolapotamon* are provided in Table 3.

**Table 3.** Morphological differences between five species of *Sinolapotamon*.

Species	*Sinolapotamoncirratum* sp. nov.	*S.patellifer* (cf. Dai 1999: fig. 78)	*S.anacoluthon* (cf. Ng and Dudgeon 1992: figs 4, 5)	*S.palmatum* (cf. Zhu et al. 2010: figs 6–9)	*S.auriculatum* (cf. Zhu et al. 2010: figs 1–4)
**Flagellum of exopod of third maxilliped**	slightly longer than width of merus (Fig. 4B)	slightly shorter than width of merus	exceeding width of merus	slightly longer than width of merus	shorter than width of merus
**Anterolateral margin of carapace**	distinctly cristate, lined with approximately 20 granules (Fig. 3A)	ridged, without conspicuous granules	cristate, lined with numerous small rounded granules	convex laterally, cristate, lined with fine granules	weakly convex laterally, cristate, lined with fine granules
**Ratio of subterminal segment to terminal segment of G1**	1.7 (Fig. 7A)	1.7 (Fig. 7B)	1.1 (Fig. 7C)	1.5 (Fig. 7E)	1.5 (Fig. 7D)
**Terminal segment of G1**	longitudinally extended oval (Fig. 7A)	oval (Fig. 7B)	longitudinally extended oval (Fig. 7C)	subcircular (Fig. 7E)	oval (Fig. 7D)
**ventral lobe of G1**	blunt, reaching 1/2 length of terminal segment (Fig. 7A)	sharp, reaching beyond proximal 2/3 length of terminal segment (Fig. 7B)	sharp and short, reaching 3/7 length of terminal segment (Fig. 7C)	sharp, reaching 5/6 length of terminal segment (Fig. 7E)	sharp, reaching proximal 1/2 length of terminal segment (Fig. 7D)

##### Etymology.

The new species is named *Sinolapotamoncirratum* sp. nov. because of the curled edges of the dorsal lobe of the G1. In the Latin, ‘cirratus’ means ‘curled’.

##### Ecology.

The specimens were collected from puddles in the Duqiaoshan Forest Park. These crabs live in the shallow water or under the wet stones (Fig. 8A, B).

##### Distribution.

China: Guangxi Zhuang Autonomous Region: Rong County, Yulin City.

### ﻿Phylogenetic relationships

**Figure 9.**
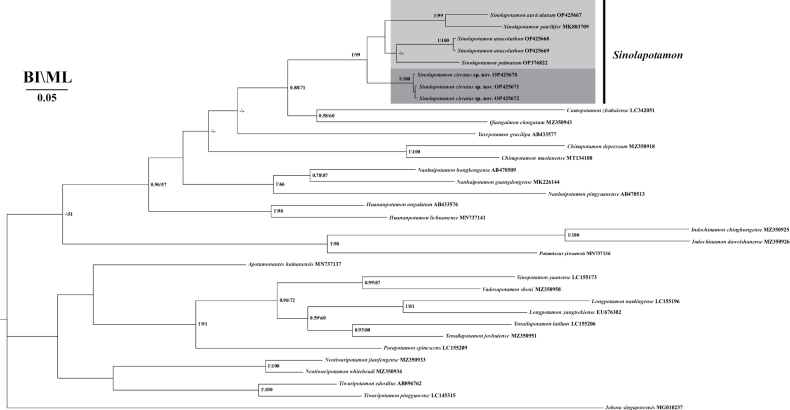
Phylogenetic tree based on the COX1 gene. Topologies and branch lengths were obtained from BI analysis. Only values >50% are displayed.

**Figure 10.**
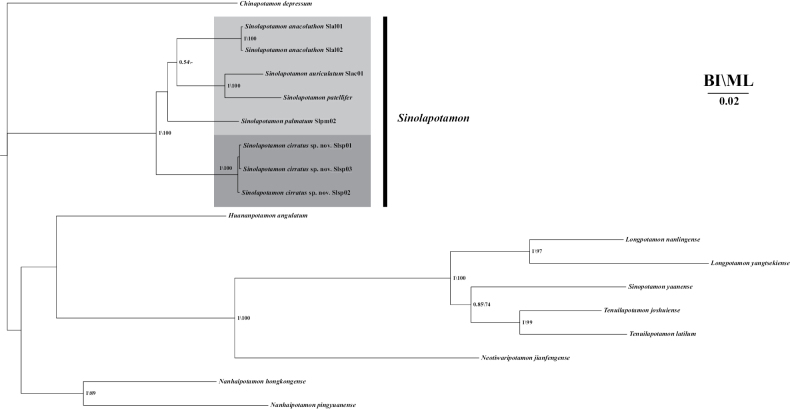
Phylogenetic tree based on three genes (COX1, 16S rRNA and 28S rRNA). Topologies and branch lengths were obtained from BI analysis. Only values >50% are displayed.

A single-gene dataset (COX1) and a 3-gene combined dataset (COX1, 16S rRNA, and 28S rRNA) were used to reconstruct the ML tree and BI tree, respectively. The topologies of the ML tree and BI tree based on the single-gene dataset and the 3-gene combined dataset were analogous. Both evolutionary trees based on the single-gene and 3-gene datasets offer strong evidence for the recognition of the new species as *Sinolapotamoncirratum* sp. nov., since it is clustered with the species of *Sinolapotamon* as a monophyletic clade. *Sinolapotamonpatellifer* and *S.auriculatum* form a sister group. Notably, *S.anacoluthon* (previously *C.anacoluthon*) is in ‘Clade *Sinolapotamon*’, which provides supporting evidence for recognizing the species in *Sinolapotamon* (Figs 9, 10). The results show that pairwise genetic distances range from 0.0600–0.1106 within the genus *Sinolapotamon*, and the genetic distances between *Sinolapotamoncirratum* sp. nov. and its congeners range from 0.0728–0.0947 (Table 4). Phylogenetic analyses, therefore, provided evidence for the identification of *Sinolapotamoncirratum* sp. nov. as a new species.

**Table 4.** Pairwise genetic distances of known species of *Sinolapotamon*.

Species	1	2	3	4
*Sinolapotamonauriculatum*	–	–	–	–
*Sinolapotamonanacoluthon*	0.0802	–	–	–
*Sinolapotamoncirratum* sp. nov.	0.0890	0.0728	–	–
*Sinolapotamonpalmatum*	0.1106	0.0692	0.0947	–
*Sinolapotamonpatellifer*	0.0600	0.0863	0.0842	0.1067

## ﻿Discussion

Previous studies on *Sinolapotamon* focused on morphological descriptions and lacked molecular evidence (Tai and Sung 1975; Ng and Dudgeon 1992; Zhu et al. 2010). For our study, we obtained sequences of the partial COX1, 16S rRNA and 28S rRNA genes of all the members of *Sinolapotamon*, thus compensating for this gap. Moreover, the taxonomic statuses of the new species and *S.anacoluthon* are demonstrated based on morphology, molecular phylogeny and geographical distribution.

Ng and Dudgeon (1992) listed the morphological differences between *Cryptopotamon* and *Sinolapotamon*, including a gently convex carapace against a strongly inflated carapace, and the extent of prominence of the epigastric and postorbital cristae. Dai (1999) stated that these differences could only be regarded as interspecific and that the fundamental types of G1 are accordant, thus considering *Cryptopotamon* as a synonym of *Sinolapotamon*. Ng et al. (2008), however, listed *S.anacoluthon* as belong to *Cryptopotamon*. We assessed the morphological differences among the five known species of *Sinolapotamon* (see Remarks above) (Table 3) and reconstructed the phylogenetic relationships in *Sinolapotamon*, in turn providing molecular evidence for transferring *C.anacoluthon* to *Sinolapotamon*. *Sinolapotamonanacoluthon* was previously recorded only from Hong Kong (Ng and Dudgeon 1992; Stanton et al. 2017), but we also collected this species in Shenzhen of the Guangdong Province. There is some geographical distance between *S.anacoluthon* (from Guangdong) and its congeners (from Guangxi). We, however, noticed that all the species of *Sinolapotamon* are distributed near the Pearl River Basin. We speculate that the Pearl River contributed to the spread of *Sinolapotamon*, but further surveys will be needed to validate this hypothesis.

## ﻿Conclusion

In this study, a new species of *Sinolapotamon* is described from the Guangxi Zhuang Autonomous Region of China, based on its morphological characteristics, especially its unique G1 among congeners, and the results of phylogenetic analyses (phylogenetic tree based on COX1 and 3-gene combined datasets). In addition, the generic position of *Cryptopotamonanacoluthon* in *Sinolapotamon* is confirmed largely on the basis of its morphology, with further evidence from the genetic data. *Sinolapotamon* is now known by five species. Based on the geographical distributions of *Sinolapotamon*, there is still possibility to discover new species in Guangxi or Guangdong.

## Supplementary Material

XML Treatment for Sinolapotamon

XML Treatment for Sinolapotamon anacoluthon

XML Treatment for Sinolapotamon cirratum

## ﻿Additional information

### Author ORCIDs

Jie-Xin Zou https://orcid.org/0000-0002-5549-2167

### Data availability

All of the data that support the findings of this study are available in the main text or Supplementary Information.

## Supplementary materials

Supplementary material 1 Phylogenetic tree (ML) based on the COX1 gene Data type phylogenetic (.mtsx file) https://binary.pensoft.net/file/861606This dataset is made available under the Open Database License (http://opendatacommons.org/licenses/odbl/1.0/). The Open Database License (ODbL) is a license agreement intended to allow users to freely share, modify, and use this Dataset while maintaining this same freedom for others, provided that the original source and author(s) are credited.Yuan-Biao Lu, Yi-Xuan Zhang, Jie-Xin Zou

Supplementary material 2 Phylogenetic tree (BI) based on the COX1 gene Data type phylogenetic (.tre file) https://binary.pensoft.net/file/861607This dataset is made available under the Open Database License (http://opendatacommons.org/licenses/odbl/1.0/). The Open Database License (ODbL) is a license agreement intended to allow users to freely share, modify, and use this Dataset while maintaining this same freedom for others, provided that the original source and author(s) are credited.Yuan-Biao Lu, Yi-Xuan Zhang, Jie-Xin Zou

Supplementary material 3 Phylogenetic tree (ML) based on 3 genes Data type phylogenetic (.mtsx file) https://binary.pensoft.net/file/861608This dataset is made available under the Open Database License (http://opendatacommons.org/licenses/odbl/1.0/). The Open Database License (ODbL) is a license agreement intended to allow users to freely share, modify, and use this Dataset while maintaining this same freedom for others, provided that the original source and author(s) are credited.Yuan-Biao Lu, Yi-Xuan Zhang, Jie-Xin Zou

Supplementary material 4 Phylogenetic tree (BI) based on 3 genes Data type phylogenetic (.tre file) https://binary.pensoft.net/file/861609This dataset is made available under the Open Database License (http://opendatacommons.org/licenses/odbl/1.0/). The Open Database License (ODbL) is a license agreement intended to allow users to freely share, modify, and use this Dataset while maintaining this same freedom for others, provided that the original source and author(s) are credited.Yuan-Biao Lu, Yi-Xuan Zhang, Jie-Xin Zou
